# Downregulation of PGM5 expression correlates with tumor progression and poor prognosis in human prostate cancer

**DOI:** 10.1007/s12672-022-00525-x

**Published:** 2022-07-12

**Authors:** Jian Sun, Fei Wang, Huihui Zhou, Chunchun Zhao, Kai Li, Caibin Fan, Jianqing Wang

**Affiliations:** 1 Department of Urology, Gusu School, The Affiliated Suzhou Hospital of Nanjing Medical University, Suzhou Municipal Hospital, Nanjing Medical University, 26 Daoqian Rd, Suzhou, Jiangsu 215000 PR China; 2 Department of Pathology, Affiliated Yuhuangding Hospital of Qingdao University, Yantai, China

**Keywords:** Prostate cancer, PGM5, Prognostic marker, Bioinformatics analysis

## Abstract

Prostate cancer (PCa) is the most common malignancy in men in developed countries. Prostate-specific antigen (PSA) remains the most widely used serum marker for prostate cancer. Here, we reported that the expression of phosphoglucomutase-like protein 5 (PGM5) is significantly lower in prostate cancer tissue. The low expression of PGM5 and its related gene signature were found to be linked to poor clinical outcome and high Gleason score. In vitro assays showed that overexpression of PGM5 significantly repressed proliferation and migration of prostate cancer cells. GO and pathway analyses showed the enrichment of genes in regulation of cell growth and migration, and pathways related in cancer. Our additional results showed that the downregulation of PGM5 is closely related to DNA methylation. Taken together, our findings provide the first evidence that PGM5 expression is associated with prostate cancer progression. These results also highlight a preclinical rationale that PGM5 represents a prognostic marker and a promising target for new therapeutic strategies in prostate cancer.

**Supplementary Information:**

The online version contains supplementary material available at 10.1007/s12672-022-00525-x.

## Introduction

In developed countries, prostate cancer is still the leading cause of cancer-related mortality in men [1]. Prostate cancer has been recognized as a common diagnostic disease due to the development of diagnostic methods, especially the introduction of PSA testing [2, 3]. However, PSA test often causes misdiagnosis or overdiagnosis, indicating that its accuracy in predicting the pathological and clinical outcome remains limited [4]. Moreover, most of the patients have indolent tumors that can be safely followed without treatment, while others with an aggressive disease course need immediate therapeutic intervention [5–7]. It is still difficult for urologists to distinguish such patients in clinic. Therefore, there is still an acute need for new biomarkers to help urologists improve the detection and prediction of prostate cancer.

Phosphoglucomutase-like protein 5 (also known as aciculin) is an enzyme encoded by the PGM5 gene. Gene functional studies show that PGM5 is similar to PGM1 but lacks enzymatic activity [8]. PGM5 is tightly associated with the actin cytoskeleton and has primarily been investigated as an adhesion protein. It also functions as a cytoskeletal component of cell–matrix and cell–cell contacts in muscle and non-muscle cells [8–10]. Recent studies showed that PGM5 has fundamental additional functions in sarcomeric development, stability and remodeling [11]. The expression and function of PGM5 in cancer, especially in prostate cancer is still largely unknown.

In the present study, we did in silico analyses based on the RNA sequencing (RNA-Seq) data from various authoritative database online to identify some new potential diagnostic and prognostic markers in prostate cancer. We finally found in multiple databases that PGM5 is a gene that is down-regulated with prostate cancer development. We further used immunohistochemistry to evaluate the expression of PGM5 protein in a wide spectrum of prostate tissues to determine its expression in situ, and evaluated the prognostic value of PGM5 in prostate cancer.

## Materials and methods

### Microarray and RNA-Seq data Information

We obtained prostate cancer and normal tissue gene expression profiles of GSE35988, GSE21032, GDS2546 and GDS1439 from the NCBI-GEO microarray database. We also downloaded the RNA-Seq dataset of prostate cancer with corresponding clinical profiles from The Cancer Genome Atlas (TCGA) database. The corresponding information related to patients, including copy number and PGM5 methylation, was obtained from the cBioPortal for Cancer Genomics website (http://www.cbioportal.org/index.do) [12]. We also digged out the relationships between PGM5 TCGA expression, DNA methylation and clinical data directly through MEXPRESS website (https://mexpress.be/) (MEXPRESS: visualizing expression, DNA methylation and clinical TCGA data).

### Publically available gene expression data sets

In this article, we used RNA and protein expression data of PGM5 in different human tissues and prostate cancer tissues through The Human Protein Atlas portal (http://www.proteinatlas.org/) [13]. All data are available directly online.

### Patient information

The cancer and para-cancer tissue samples from a group of 71 patients with prostate cancer were collected in this study. All the tissues were fixed in formalin after surgical removal. The Ethics Committee of Nanjing Medical University approved the research and written informed consents were obtained from the participants. Patients with a previous history of malignant tumors were excluded from this study. We showed the clinicopathological information of the tissues collected in Additional file 1: Table S1.

### Tissue microarrays (TMAs) and immunohistochemistry (IHC)

We collected the prostate cancer and para-cancer tissues to construct TMAs as previously described [14]. IHC was performed as described elsewhere [15, 16]. In brief, slides were deparaffinized and heated in citrate buffer pH6 for antigenic retrieval. The primary antibody was PGM5 (Novus). Immunohistochemistry was performed using the streptavidin–biotin-peroxidase method with diaminobenzidine as the chromogen (KitLSAB, Dakocytomotion, Glostrup, Denmark). Negative controls were obtained after omission of the primary antibody or incubation with an irrelevant antibody.

PGM5 staining was simultaneously examined by two independent observers (including one pathologist), and a consensus score was reached for each core. A positive reaction for PGM5 was scored in four grade categories depending on the intensity of the staining, i.e., 0, 1, 2 and 3, and the percentage of PGM5-positive cells was also scored in four groups: 0 (0%), 1 (1 to 33%), 2 (34 to 66%) and 3 (67 to 100%). In cases with discrepancies between duplicated cores, the higher score of the two tissues was taken as the final score. The sum of the intensity and percentage scores was used as the final staining score. The staining pattern was defined as follows: 0, negative; 1 to 2, weak; 3 to 4, moderate; and 5 to 6, strong [17].

### Gene set enrichment analysis (GSEA)

We analyzed differences in biological function annotations of gene mRNA expression levels between PGM5 up- and down-regulated patients by GSEA v3.0. The results indicated the effect of PGM5 expression on various biological function gene sets in prostate cancer patients. The number of permutations was set at 10. In this analysis, we used the following values as cutoff: nominal P-value cutoff of 0.05; false discovery rate (FDR q-val) < 0.25.

### Cell culture

We obtained LNCaP, PC-3 and DU145 cells from ATCC (Bethesda, USA). All cells were maintained in RPMI-1640 supplemented with 10% FBS and antibiotics (0.1 mg/ml streptomycin and 100 units/ml penicillin) as previous [18]. All cell lines used in our study were authenticated by STR profiling and tested for mycoplasma contamination.

### Constructs and construction of stable cell lines

We used pPB-CAG-EBNXN vector (Sanger Institute) to deal with constructs and pPB-CAG-ires-Pac was generated as previously described [18, 19]. We ligatied full length PGM5 into the multiple-cloning sites (MCS) of pPB-CAG-ires-Pac to generate pPB-CAG-PGM5-ires-Pac. Control and PGM5 overexpression stable cells were obtained as previously described and all stable cell lines were selected and identified by western blotting [18].

### Antibodies and immunoblotting

In western blotting, cells were lysed in 1 × SDS loading buffer (50 mM Tris–HCl pH6.8, 10% glycerol, 2% SDS, 0.05% bromophenol blue and 1% 2-mercaptoethanol). Antibodies were listed as follows: anti-PGM5 antibody (NBP2-62654, Novus Biologicals) and anti-ACTIN (ab8227, Abcam).

We did immunoblot as previously described [18]. In brief, all proteins were separated by SDS–PAGE and were transferred to polyvinylidene difluoride membranes (Millipore). HRP-labeled secondary antibodies and enhanced chemiluminescence system was used for signal detection. Protein was visualized using ChemiDoc XRS chemiluminescence detection and imaging system (Bio-Rad Laboratories) or KODAK film machine.

### Cell proliferation (MTS) assay

Cell proliferation (MTS) assay was performed as described previously [20]. In brief, all cells were seeded at 4000 cells/well (0.1 ml) in 96-well plates and were incubated overnight at 37 °C for 5–6 days. In each indicated time point, cells were incubated with 20 μl of CellTiter 96 AQueous One solution reagent MTS (Promega) in 100 μl of RPMI-1640 for one hour. Cell number was then estimated using a microtiter plate reader (Bio-tek).

### Identification of differentially expressed genes (DEGs)

EdgeR for examining differential expression of RNA-Seq count data as previously described [21, 22]. DEGs were identified with the following criterion: |fold change (FC)|≥ 2; the P-value and FDR < 0.05. The DEGs were used for further bioinformatics analysis. A heat map of top 100 genes and volcano plot of the DEGs were drawn.

### Cell migration assays

We used Transwell (Corning) system in 24-well tissue culture plates to deal with cell migration assays as described previously [18].

### Functional annotation and pathway enrichment analysis

We used the database for annotation, visualization and integrated discovery (DAVID) website to undergo GO analysis and KEGG analysis [23], while specifying a P-value < 0.05 for statistical significance.

### Online ChIP-seq analysis

Online ChIP-seq data of different Histone methylation in prostate cancer cells were obtained by UCSC browser from Cistrome Data Browser online [24].

### Statistical analysis

The results are presented as the mean values ± SEM. The association between PGM5 staining and the clinicopathologic parameters of the prostate cancer patients were evaluated by a χ2 test. Differences between groups were estimated using Student’s t test. For analysis of the association between PGM5 expression and biochemical recurrence, we used Kaplan–Meier method and log-rank test by Graphpad. Receiver operating characteristic (ROC) curve analysis was also dealt using Graphpad. Areas under the curves (AUCs) were used to evaluate the diagnostic value of each marker combination. An AUC greater than 0.9 indicated excellent diagnostic efficacy. An AUC between 0.7 and 0.9 indicated good diagnostic efficacy. FDR in edgeR and GSEA were adjusted for multiple testing with the Benjamini–Hochberg procedure to control FDR, respectively [25, 26]. A value of P < 0.05 was considered statistically significant. All the statistical analyses were conducted with Graphpad and R 3.3.0.

## Results

### Identification of differentially expressed genes (DEGs)

**Fig. 1:**
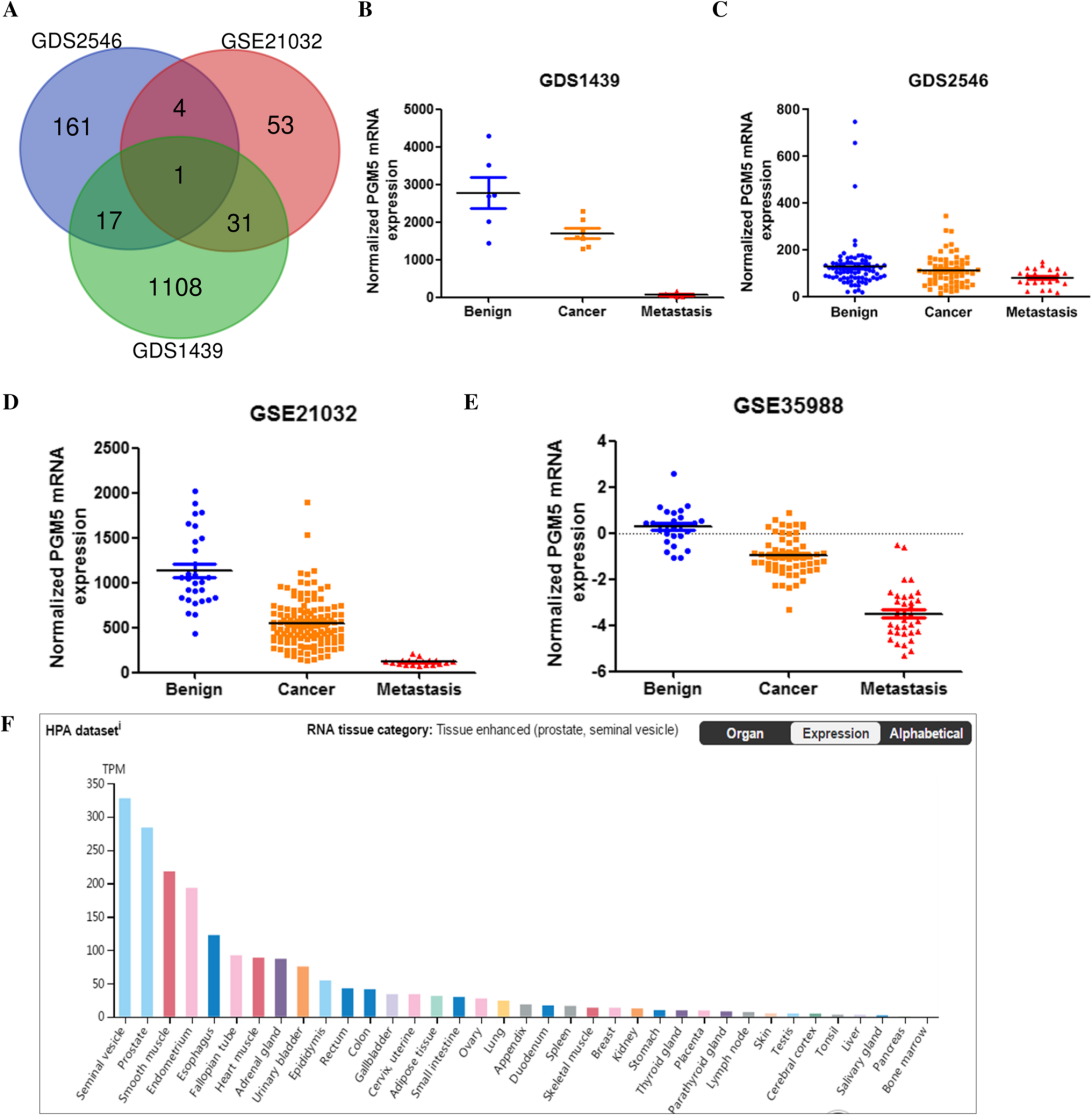
Associations between PGM5 expression and prostate cancer progress.**A** Identification of only one common DEGs from the 3 cohort profile data sets (GSE21032, GDS1439, and GDS2546) using an online tool Calculate and draw custom Venn diagrams (http://bioinformatics.psb.ugent.be/webtools/Venn/). **B**–**D** PGM5 mRNA level was downregulated in primary and metastatic prostate cancer in datasets GDS1439, GDS2546 and GSE21032. **E** Data from GSE35988 confirmed the low PGM5 mRNA level in primary and metastatic prostate cancer. **F** Data from The Human Protein Atlas portal show PGM5 expression in different tissues

To identify the DEGs, we downloaded and analyzed the prostate cancer and normal tissue gene expression profiles of GSE21032, GDS2546 and GDS1439 from the NCBI-GEO microarray database. Based on the in silico analysis, using P < 0.05 and fold change (FC) ≥ 2.0 or ≤ 0.5 as criteria. After integrated bioinformatical analysis, only one gene, PGM5, was identified from the three profile datasets above (Fig. 1 A). We also showed the epxression patterns of PGM5 in the three datasets in Fig. 1 B-D, respectively. To confirm PGM5 expression, we analyzed PGM5 expression in another dataset, GSE35988. The same result showed PGM5 downregulation in GSE35988 (Fig. 1 E). To further investigate the mRNA expression of PGM5, we explored The Human Protein Atlas portal and found relatively high expression of PGM5 in normal prostate tissue (Fig. 1 F). Thus, our findings above indicated that PGM5 expressed much lower in prostate cancer tissue, which makes it a potential biomarker for prostate cancer development.

### Clinical impact of PGM5 expression on prostate cancer progression

**Fig. 2:**
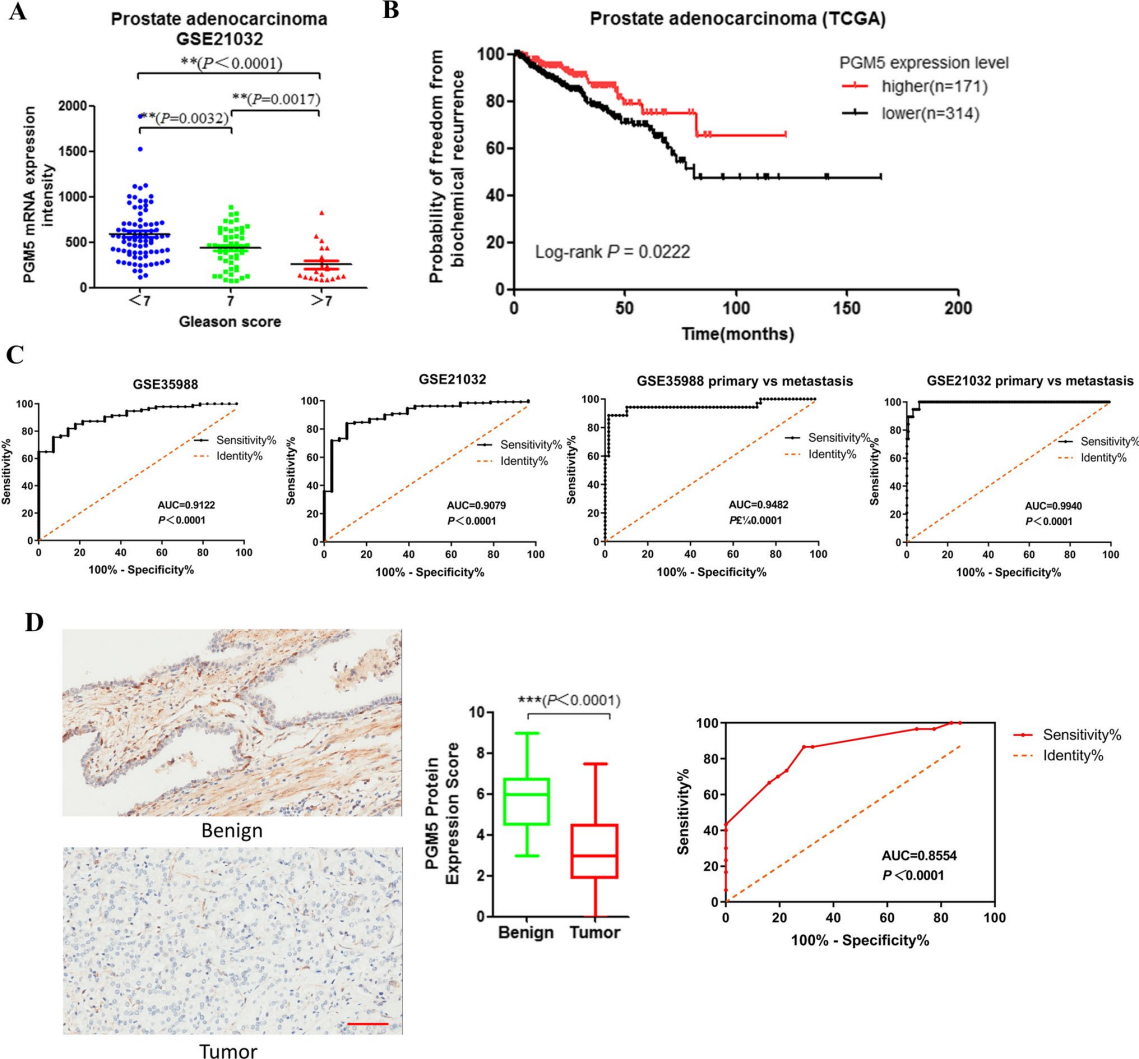
PGM5 expression is associated with prostate cancer progress severity and prognosis. **A** Correlation between PGM5 expression and Gleason score. **B** Kaplan–Meier survival curves for prostate cancer patients stratified by PGM5 expression. C ROC curves of PGM5 expression in prostate cancer patients. **D** Representative immunohistochemistry of PGM5 on benign prostatic epithelia and prostate cancer tissues

We next determined whether PGM5 expression influences prostate cancer progression. We first investigated the relationship between PGM5 expression and the clinic pathological characteristics in the TCGA prostate cancer dataset using cBioPortal for Cancer Genomics [20]. Results showed that the downregulation of PGM5 appeared to significantly correlate with higher Gleason score, but not the tumor stage (Fig. 2 A and Table 1). More importantly, we found that patients with a lower PGM5 level in tumor tissues showed much poorer prognosis (Fig. 2 B). All these results revealed that the low level of PGM5 positively correlates with advanced stage, increased metastasis, and poor prognosis in PCa patients.

**Table 1:** Clinical Characteristics of PCa Patients and PGM5 Expression in TCGA

Characteristics	Expression of PGM5 in PCa		*P* Value
	Low	High	
Age, years			
≤ 65	232	119	0.3472
> 65	86	54	
Gleason score			
< 7	22	23	**0.009**
7	152	92	
> 7	144	58	
Tumor stage			
≤ T2a	7	6	0.4279
T2b	8	2	
≥ T2c	299	162	
N stage			
N0	222	120	0.0796
N1	58	19	
M stage			
M0	288	161	0.1961
M1	3	0	

P < 0.05 was considered statistically significant. Chi-square test was used

To analyze the value of PGM5 in the diagnosis and disease prognosis of prostate cancer, we performed ROC curve analysis for PGM5 in healthy individuals and in PCa patients, or in primary cancer and in metastatic cancer. Results in Fig. 2 C showed that in different data sets, PGM5 was useful for prostate cancer diagnosis in patients. Moreover, the expression level of PGM5 was also useful for distinguish metastatic patients from primary patients. All the AUCs were greater than 0.9.

In addition to the RNA level, we also detected PGM5 expression in protein level. We examined PGM5 protein expression by IHC in prostate cancer and matched para-cancer tissues. As shown in Fig. 2 D, immunoreactivity of the PGM5 protein was observed in cell cytoplasm. After analyzing PGM5 expression pattern in prostate cancer tissues, we found that in cancer tissues, 4 cases were strong (5–6 points), 19 cases were moderate (3–4 points), 37 cases were weak (1–2 points), and 11 cases were negative (0 point), while in para-cancer tissues, we found that 33 cases were strong, 24 cases were moderate, 14 cases were weak, and 0 case was negative. Data from HPA showed the similar result (Additional file 2: Figure S1A). Our data indicated that prostate cancer tissues express much lower PGM5 in compared with benign tissues.

### PGM5 could suppress prostate cancer proliferation and migration

**Fig. 3:**
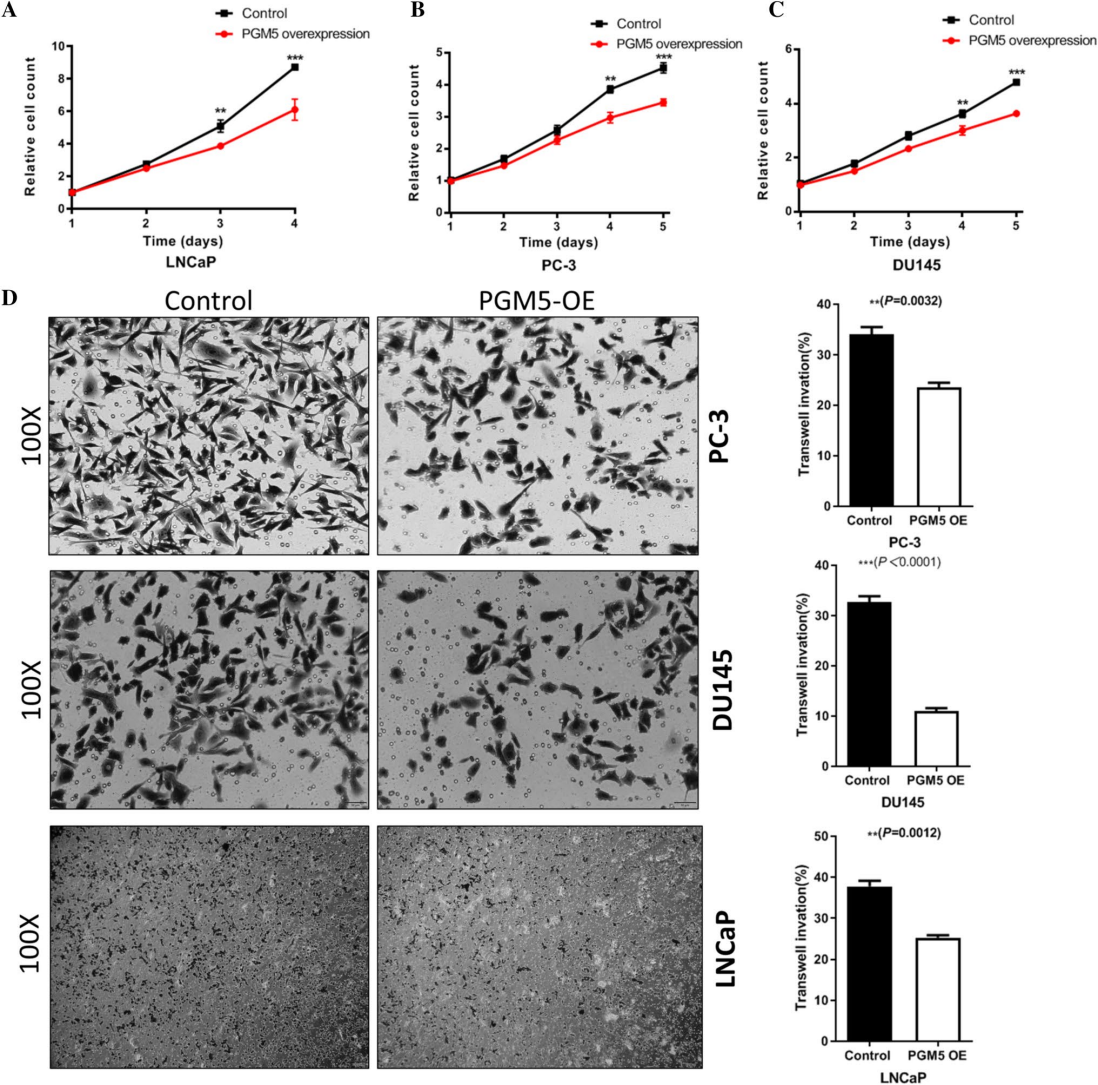
PGM5 suppresses prostate cancer cell proliferation and migration. **A** Cell proliferation was measured at the indicated time points in LNCaP cells (** *P* < 0.01, *** *P* < 0.001, N = 3). **B** Cell proliferation was measured at the indicated time points in PC-3 cells (** *P* < 0.01, *** *P* < 0.001, N = 3). **C** Cell proliferation was measured at the indicated time points in DU145 cells (** *P* < 0.01, *** *P* < 0.001, N = 3). **D** Transwell assay analyses of the indicated cell lines (N = 3)

To further investigate the role of PGM5 in prostate cancer progression, we overexpressed PGM5 in LNCaP, DU145 and PC-3 prostate cancer cell lines (Additional file 2: Figure S1B). We determined the effect of PGM5 on prostate cancer cell proliferation and migration. In vitro assays showed that up-regulation of PGM5 repressed cell proliferation and migration (Figs. 3 A-D). These results above suggested the tumor suppressor role of PGM5, and also confirmed the prognostic role of PGM5 in prostate cancer.

### Functional enrichment analyses of PGM5 correlated genes

**Fig. 4:**
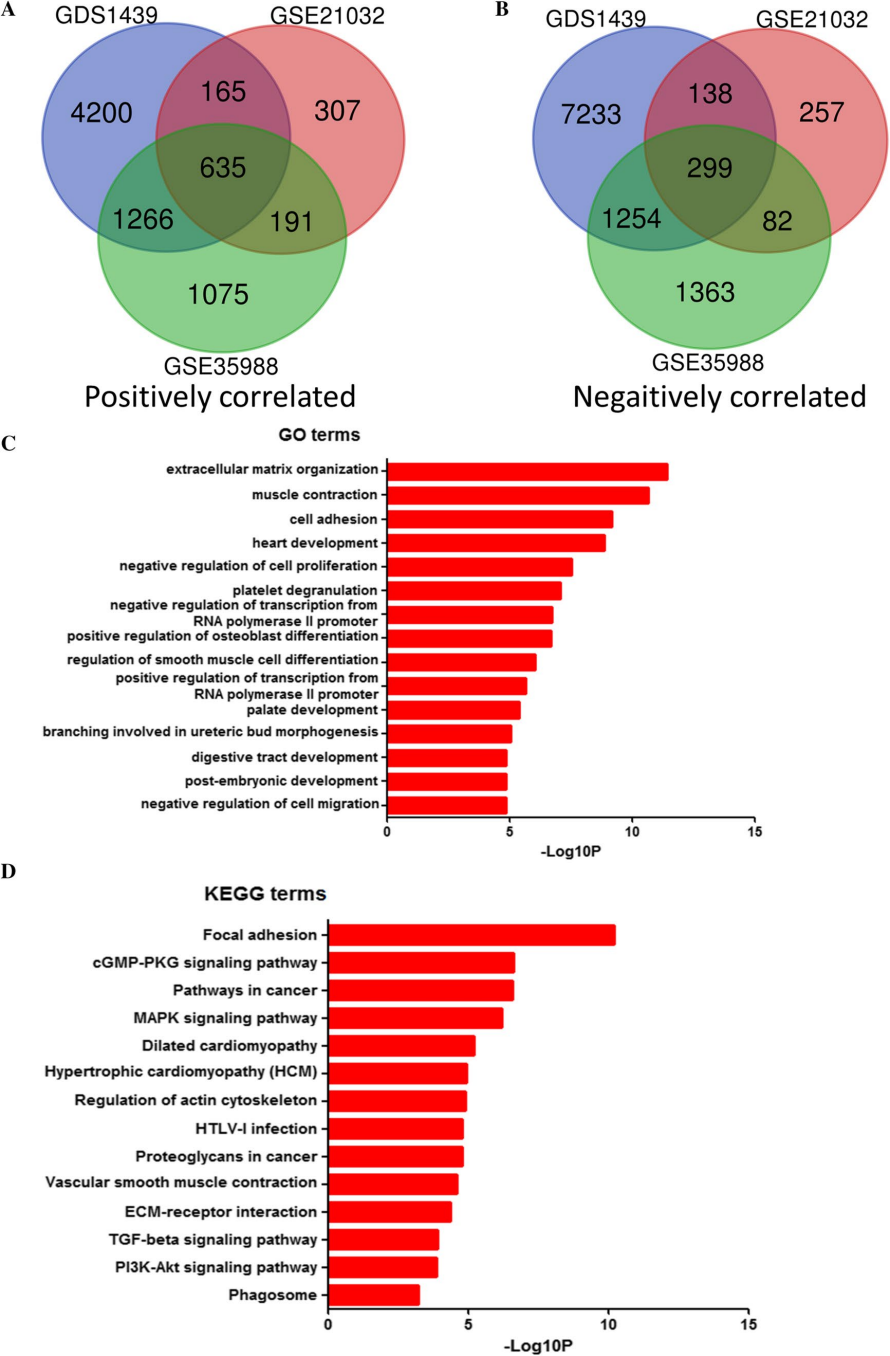
DAVID enrichment results of correlated genes of PGM5. **A** Bioinformatic analysis showed 635 positively correlated genes in GSE21032, GSE35988 and GDS1439. **B** Bioinformatic analysis showed 299 negatively correlated genes in the three datasets above. **C** The GO enrichment terms of positively correlated genes. **D** The KEGG pathway analysis of positively correlated genes

To further explore the mechanisms of PGM5 in prostate cancer and find out some testable hypotheses, we did the correlation analysis using the datasets from NCBI-GEO above. Data of GSE35988, GSE21032 and GDS1439 were recruited for the analysis. Correlation coefficient was calculated and the cutoff was 0.4/-0.4. After integrated bioinformatical analysis, total of 934 consistently correlated expressed genes were identified from the three profile datasets, including 635 positively correlated genes and 299 negatively correlated genes (Fig. 4 A, B, Additional file 3: Table S2).

In order to analyze the correlated genes at the functional level, we submitted the two groups of genes separately online for further analyses with DAVID. GO and KEGG analyses showed enrichment of positively correlated genes, while negatively correlated genes showed no significant enrichment. We showed the results of GO analysis of positively correlated genes in Fig. 4 C. These results indicated that most of the positively correlated genes were significantly enriched in cell migration, proliferation and differentiation. We showed the results of signaling pathway enrichment in Fig. 4 D. KEGG pathway analysis showed that positive correlation genes had common pathways related in cancer development.

To further investigate the effect of PGM5 downregulation on the development of prostate cancer, we analyzed the effects of PGM5 downregulation on various gene sets by the GSEA. Both data from GSE35988 and TCGA datasets were used to confirm the results. GSEA results of GSE35988 and TCGA dataset were shown in Additional file 4: Figure S2, respectively. This suggested that PGM5 downregulation may affect disease progression and prognosis in prostate cancer patients by influencing cell cycle, DNA repair and multiple oncologic pathways, which could be further explored and confirmed in future analysis.

### PGM5 promoter methylation correlates with PGM5 downregulation

**Fig. 5:**
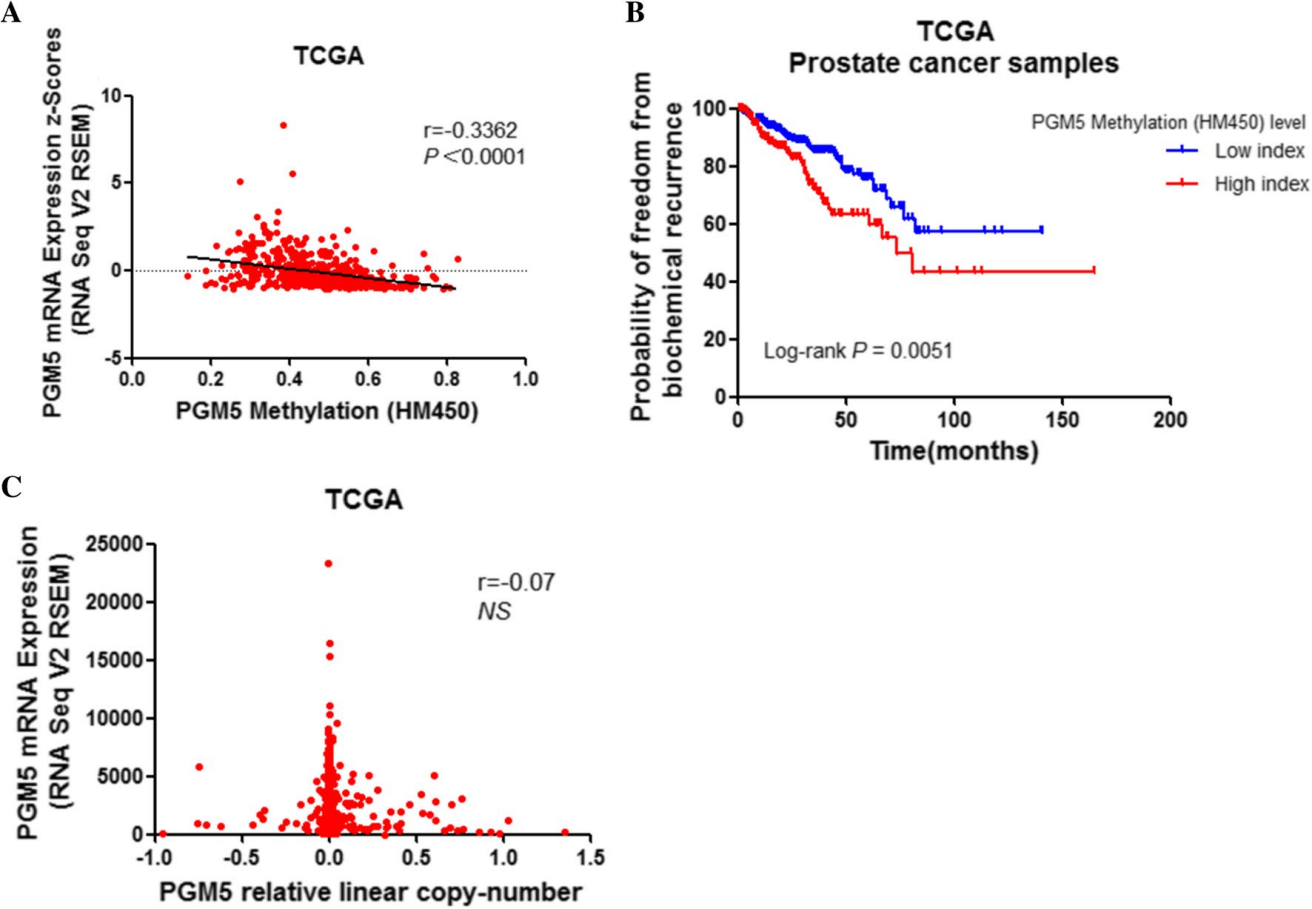
PGM5 mRNA expression correlated with PGM5 promoter methylation level. **A** PGM5 mRNA expression is inversely correlated with PGM5 methylation level in prostate samples from TCGA dataset. **B** Kaplan–Meier survival curves for prostate cancer patients stratified by PGM5 methylation level. C PGM5 mRNA expression is not correlated with PGM5 copy number in prostate samples from TCGA dataset

Considering the important role of PGM5 downregulation in prostate cancer disease progression, we further analyzed the mechanisms of decreased PGM5 expression and conducted preliminary investigation. DNA methylation, especially promoter methylation, plays a key role in transcriptional regulation and always contributes to silencing gene expression. To test the hypothesis that the methylation of DNA could play a role in regulating PGM5 expression in prostate cancer, we explored the correlation between PGM5 methylation and PGM5 expression in TCGA dataset by cBioPortal for Cancer Genomics. Our database analysis showed that high PGM5 methylation level is correlated with low PGM5 expression level in prostate tumors and with poor prognosis in prostate cancer patients (Fig. 5 A, B). What’s more, we confirmed the result in another online data visualization tool, MEXPRESS (https://mexpress.be). We found a much lower level of methylation in CpG island in normal tissues than that of prostate cancer tissues, which also suggests that DNA methylation is likely to be one of the important mechanisms for downregulation of PGM5 expression (Additional file 5: Figure S3A).

Further more, we also detected other potential mechanisms for PGM5 downregulation. We first examined whether DNA copy number alteration influenced PGM5 expression. Data from TCGA indicated that DNA copy number alteration did not corelate to PGM5 mRNA expression level, thus ruling out this possibility (Fig. 5 C). We next investigated whether histone methylation caused a decrease in PGM5 expression. To test this hypothesis, we explored the public ChIP-seq (chromatin immunoprecipitation assays with sequencing) data of histone methylation markers in prostate cancer cell lines [36]. We noticed no binding events of either active or repressive histone methylation marks (e.g. H3K4me3 and H3K27me3) in the DNA region of PGM5 in LNCaP-abl or LNcaP cell lines (Additional file 5: Figure S3B), suggesting that histone methylation does not primarily affect the expression of PGM5.

## Discussion

In the present study, our results demonstrate that PGM5 expression is decreased in primary and metastatic prostate cancer by bioinformatic analyses and immunohistochemical assessment, which indicate a novel role for PGM5 as an important diagnostic and prognostic marker in prostate cancer.

Current markers for diagnosis and prognosis of PCa, such as PSA test, tumor stage and Gleason score, have been used as the main methods to diagnose or predict prostate cancer in the past few decades [27]. However, such methods are limited in their accuracy and prognostic ability [28]. A typical example is that the single test of PSA also caused a high rate of misdiagnosis or overdiagnosis [4]. Therefore, the development of additional prognostic markers is urgent. Our results provide a new sight into discovering a new diagnostic and prognostic marker in addition to PSA. We summarized the gene expression data of several authoritative prostate cancer clinical sequencing databases and found PGM5 downregulation in tumor tissues in all datasets. Further analysis indicated the tumor suppressor role of PGM5 in prostate cancer, which was also confirmed by in vitro assays and IHC in tumor tissues from our patients.

Radical prostatectomy is still the best treatment for clinically localized prostate cancer [29]. Recurrence of the disease in men after radical prostatectomy suggests that undetected disease may have spread beyond the prostate gland before surgery [30, 31]. Increase the detection rate of this part of the patients will assist in designing a more effective therapeutic strategy, including aggressive treatment. In this study, our results indicate that patients with lower PGM5 expression are more prone to poor prognosis, and may benefit from early intervention.

We also digged out the main biologic pathways implicated in this process. Correlation analysis was performed in several clinical datasets to find out the primary genes that were consistently expressed in association with PGM5 expression. Through the analysis of these positively/ negatively corelated genes, we found that several biologic processes related to oncogenesis were enriched. Signaling pathway analysis also showed enrichment in pathways that have been proven to be implicated in cancer development. In addition to the findings above, GSEA analysis also found that PGM5 also implicated in DNA repair. Previous researches have shown that metastatic castration-resistant prostate cancer have genomic aberrations that interfere with DNA repair [32, 33]. Poly (adenosine diphosphate [ADP]–ribose) polymerase (PARP) is involved in multiple aspects of DNA repair, and PARP inhibition has been shown to exert antitumor activity in sporadic cases of metastatic, castration-resistant prostate cancer by dealing with DNA-repair defects in tumor cells [34]. All these results above indicate many testable hypotheses that these biology processes and pathways might implicate in prostate cancer initiation and progression. PGM5 may influence cell proliferation, migration and differentiation through mutiple signaling pathways, thus promoting or inhibiting cancer progression, which needs further validation via molecular biologic experiments.

As for how PGM5 downregulated in prostate cancer initiation and development, our results suggest DNA methylation might be the potential mechanism. DNA methylation is an important process needed for normal development. It has been quite clear that the methylation of gene promoters often leads to repression of transcription [35]. In prostate cancer, critical oncogenes or tumor suppressor genes were methylated or de-methylated in cancer progression by mutiple mechanisms. Changes in DNA methylation influence gene expression and have been regarded as potential therapeutic targets [36, 37]. Methylation of PGM5 might be a potential prostate cancer research and drug target, which also needs further molecular biological experiments to validate.

Our study contained several limitations. We utilized a series of online RNA-seq data to explore the role of PGM5 in prostate cancer by bioinformatics analysis. Most of the results are preliminary, such as the mechanisms of PGM5 downregulation in prostate cancer. In the following research, we will use molecular biological experiments to validate our results.

## Conclusions

In conclusion, we found the expression of PGM5 is significantly lower in prostate cancer tissue. Lower expression of PGM5 was found to be linked to high Gleason score and poor clinical outcome. Moreover, downregulation of PGM5 is closely related to DNA methylation. Taken together, our findings provide the first evidence that PGM5 expression is associated with prostate cancer progression, which suggest PGM5 as a promising prognostic marker and therapeutic target in prostate cancer.

## Supplementary Information

**Additional file 1: Table S1.** Clinical characteristics of prostate cancer patients.

**Additional file 2: Figure S1.** PGM5 expression in HPA portal and overexpression effect in prostate cancer cells. **A** IHC data from The Human Protein Atlas portal show PGM5 expression is lower in prostate cancer tissues than in normal tissues. **B** Western blot analyses of PGM5 expression in the indicated cell lines with overexpression.

**Additional file 3: Table S2.** Correlated genes of PGM5 identified from 3 datasets.

**Additional file 4: Figure S2.** GSEA results of PGM5 expression in prostate cancer patients in GSE35988 (**A**) and TCGA (**B**).

**Additional file 5: Figure S3.** PGM5 promoter methylation level in online datasets. A PGM5 promoter methylation in MEXPRESS (https://mexpress.be). **B** Online ChIP-seq data of present Histone methylation marks in prostate cancer cells.
