# Supplementary figures and images for: Downregulation of PGM5 expression correlates with tumor progression and poor prognosis in human prostate cancer
Source: Discov Oncol. 2022 Jul 12;13:63. doi: 10.1007/s12672-022-00525-x (PMC9276915; doi:10.1007/s12672-022-00525-x)

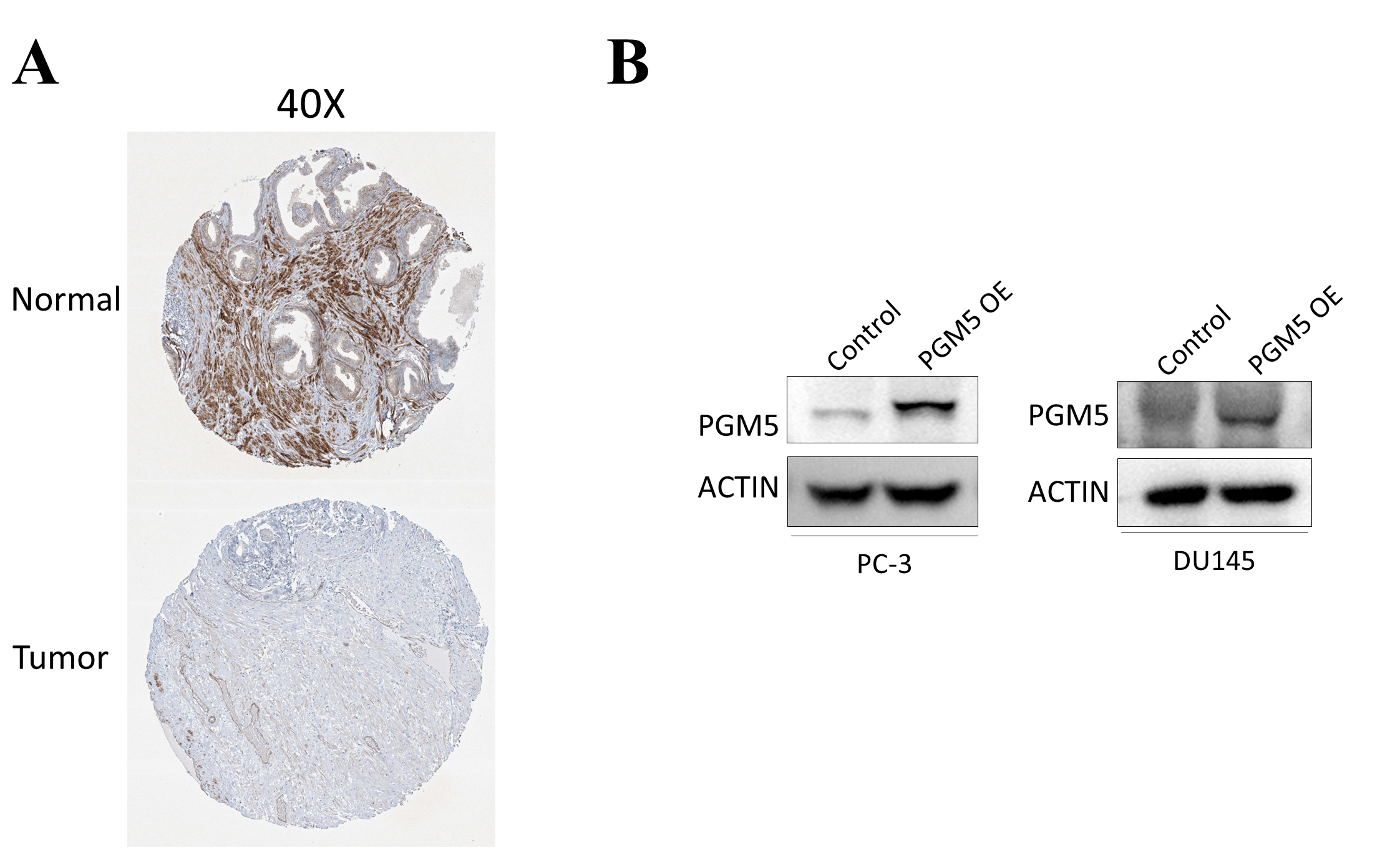

Supplement: Supplementary file 2 — Additional file 2: Figure S1. PGM5 expression in HPA portal and overexpression effect in prostate cancer cells. A IHC data from The Human Protein Atlas portal show PGM5 expression is lower in prostate cancer tissues than in normal tissues. B Western blot analyses of PGM5 expression in the indicated cell lines with overexpression. [file 12672_2022_525_MOESM2_ESM.tif]

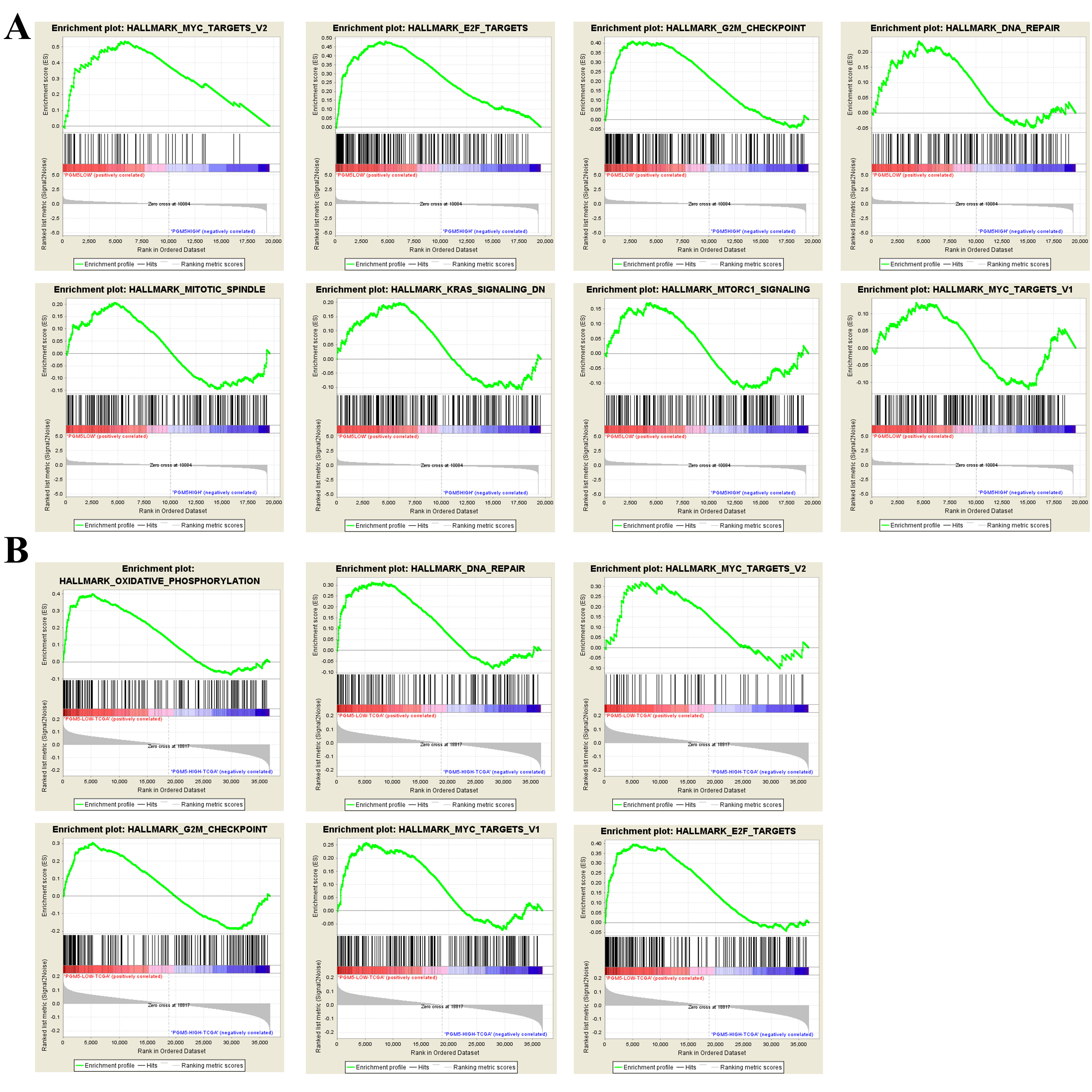

Supplement: Supplementary file 4 — Additional file 4: Figure S2. GSEA results of PGM5 expression in prostate cancer patients in GSE35988 (A) and TCGA (B). [file 12672_2022_525_MOESM4_ESM.tif]

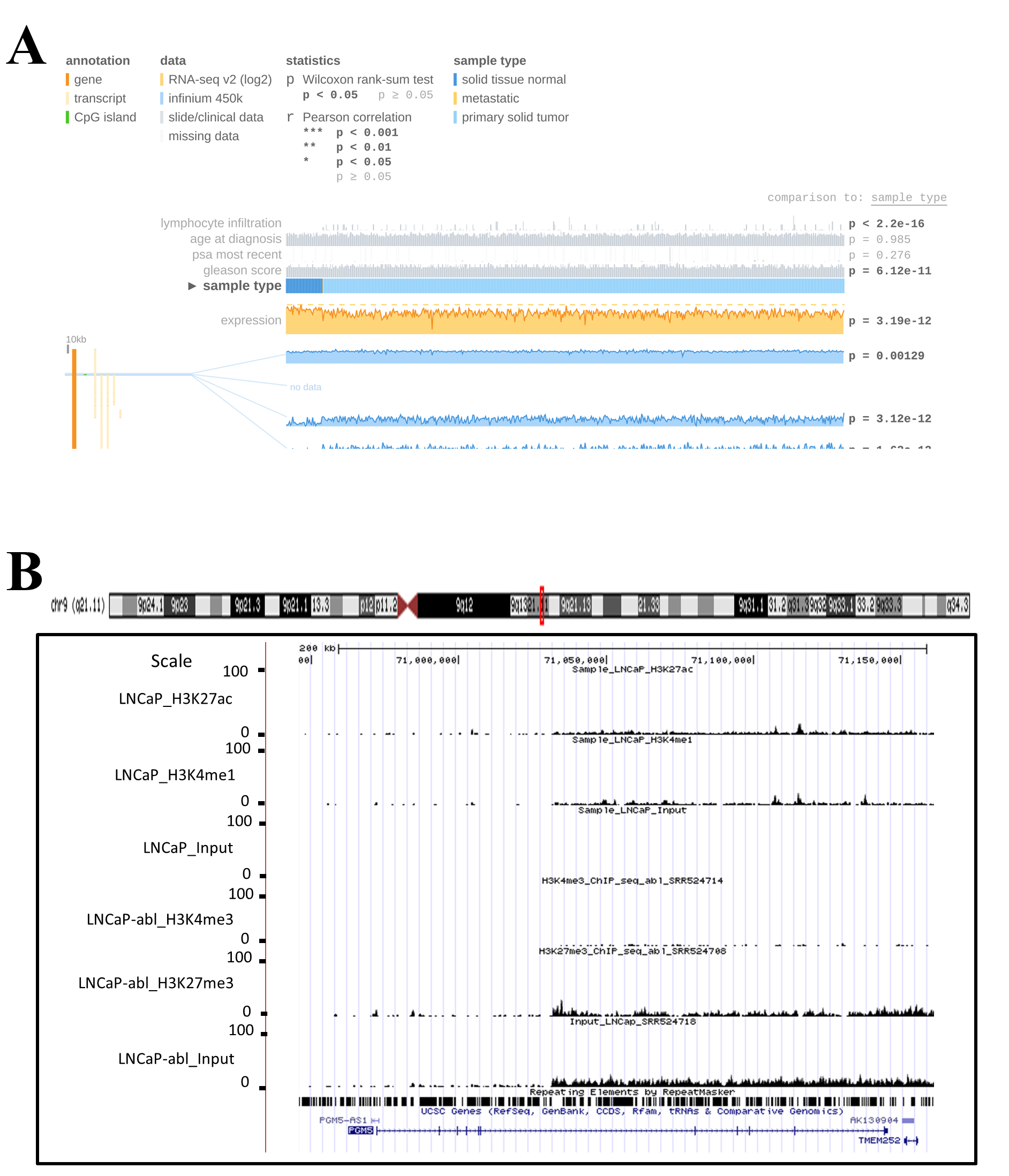

Supplement: Supplementary file 5 — Additional file 5: Figure S3. PGM5 promoter methylation level in online datasets. A PGM5 promoter methylation in MEXPRESS (https://mexpress.be). B Online ChIP-seq data of present Histone methylation marks in prostate cancer cells. [file 12672_2022_525_MOESM5_ESM.tif]
